# Simplifying the detection and monitoring of protein glycosylation during in vitro glycoengineering

**DOI:** 10.1038/s41598-023-27634-z

**Published:** 2023-01-11

**Authors:** Matthew J. Saunders, Robert J. Woods, Loretta Yang

**Affiliations:** 1 Lectenz Bio, 111 Riverbend Rd, Athens, GA 30602 USA; 2 Complex Carbohydrate Research Center, University of Georgia, 315 Riverbend Rd, Athens, GA 30602 USA

**Keywords:** Assay systems, Carbohydrates

## Abstract

The majority of mammalian proteins are glycosylated, with the glycans serving to modulate a wide range of biological activities. Variations in protein glycosylation can have dramatic effects on protein stability, immunogenicity, antibody effector function, pharmacological safety and potency, as well as serum half-life. The glycosylation of therapeutic biologicals is a critical quality attribute (CQA) that must be carefully monitored to ensure batch-to-batch consistency. Notably, many factors can affect the composition of the glycans during glycoprotein production, and variations in glycosylation are among the leading causes of pharmaceutical batch rejection. Currently, the characterization of protein glycosylation relies heavily on methods that employ chromatography and/or mass spectrometry, which require a high level of expertise, are time-consuming and costly and, because they are challenging to implement during in-process biologics production or during in vitro glycan modification, are generally performed only post-production. Here we report a simplified approach to assist in monitoring glycosylation features during glycoprotein engineering, that employs flow cytometry using fluorescent microspheres chemically coupled to high-specificity glycan binding reagents. In our GlycoSense method, a range of carbohydrate-sensing microspheres with distinct optical properties may be combined into a multiplex suspension array capable of detecting multiple orthogonal glycosylation features simultaneously, using commonplace instrumentation, without the need for glycan release. The GlycoSense method is not intended to replace more detailed post-production glycan profiling, but instead, to complement them by potentially providing a cost-effective, rapid, yet robust method for use at-line as a process analytic technology (PAT) in a biopharmaceutical workflow or at the research bench. The growing interest in using in vitro glycoengineering to generate glycoproteins with well-defined glycosylation, provides motivation to demonstrate the capabilities of the GlycoSense method, which we apply here to monitor changes in the protein glycosylation pattern (GlycoPrint) during the in vitro enzymatic modification of the glycans in model glycoproteins.

## Introduction

Glycoproteins exists not as single molecules, but as ensembles of proteoforms (glycoforms), in which a range of glycan structures may be present at any given glycosylation position (glycosite) in the polypeptide. This variation arises because during glycan processing is controlled in vivo by interactions with enzymes as the glycoprotein passes through the endoplasmic reticulum and Golgi apparatus, as well as by subsequent exposure to endogenous glycosidases.

Glycoprotein bioactivity is known to depend heavily on the composition of the ensemble of glycans (the glycosylation state), with some glycoforms being more or less bioactive than others^1–5^. For example, reduction in the level of sialic acid in a glycan often lowers the serum half-life of the glycoprotein^6^, while the inadvertent introduction of non-endogenous glycan structures, such as α-galactose (α-Gal), can trigger anaphylaxis^7^. It is not surprising, therefore, that the US Food and Drug Administration (FDA) and the European Medicines Agency (EMA) require that the glycosylation patterns of all therapeutic glycoproteins be extensively characterized and maintained within the specifications defined at the time of licensure^8,9^. This requirement places an essential, but challenging burden on the production of glycoprotein biologics, such as monoclonal antibodies (mAbs), hormones, etc.^10^.

Maintaining a consistent glycosylation profile batch to batch or during scale-up is notoriously difficult to achieve when glycoproteins are produced in bioreactors, due to the sensitivity of the relevant enzymatic activities to subtle variations in media composition, dissolved gas concentrations, temperature, and even reactor volume^11–13^. For this reason, there is a growing interest in the use of in vitro or chemoenzyatic glycoengineering approaches that attempt to generate uniform glycosylation or hyperglycosylation by post-production treatment of the expressed glycoprotein with a succession of glycosidases that trim the glycans back to a uniform core structure, followed by glycan extension using sequential glycosyltransferases^14–16^. Moreover, as patents on originator biologics expire, there is growth in the development of generic biosimilars, such as mAbs, which are interchangeable with the already-approved biologic in terms of efficacy, safety, purity, and potency^17^. Although biosimilars can readily be produced with identical amino acid sequences as the originator product, it is extremely challenging to ensure that the glycosylation states are comparable^18^.

In typical glycosylation analyses (glycoprofiling), the glycans are released from the purified glycoprotein by treatment with one or more enzymes before being structurally characterized using mass spectroscopy-based methods or by chromatography employing reference standards. While the gold standard, more advanced glycoprofiling of this type may require additional training in the relevant technologies and significant capital expenses. For these reasons, in both industrial and academic settings, glycoprofiling is frequently performed at dedicated core facilities. These approaches permit the complete identification of all of the glycans in a given sample as well as their relative abundance. Yet the time required for the analysis often makes in-process monitoring of glycosylation impractical. Thus despite the fact that variations in glycosylation can lead to batch rejection, glycosylation is not routinely monitored at-line during glycoprotein cell cultivation, nor during the optimization of cell culture conditions, nor during in vitro glycoengineering. A statistically robust, rapid, and cost-effective analytical method for in-process monitoring of glycosylation would both substantially lower the cost of commercially produced biologics, and enhance the scientific community’s ability to deduce relationships between glycan structure and biological function. Such a capability would also be uniquely useful in the early development stages of new biologics and biosimilars, by facilitating the rapid optimization of process parameters and conditions that affect the glycosylation state.

**Figure 1:**
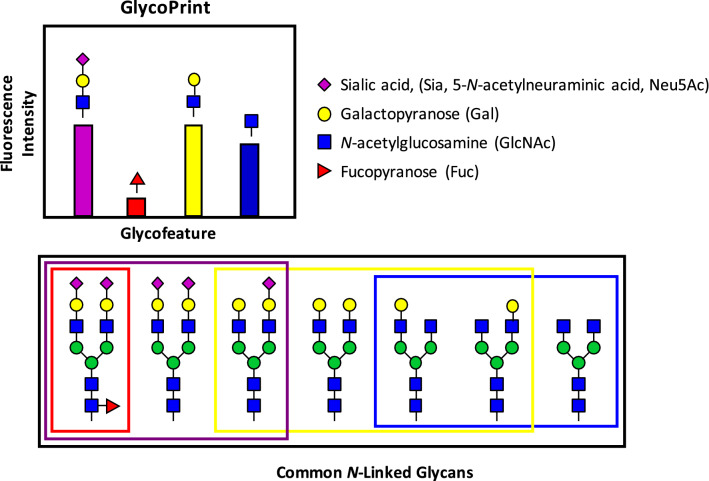
A hypothetical GlycoPrint from an ensemble of 7 typical *N*-linked glycans with unique glycosylation elements grouped by color and monosaccharides shown in SNFG notation^51^. The choice of detection element (lectin, antibody, or other carbohydrate-specific reagent) determines whether the glycofeature will comprise a single monosaccharide or larger oligosaccharide. A GlycoPrint is a representation of the aggregate signal for each glycofeature.

Here we introduce an orthogonal approach to traditional glycoprofiling, called GlycoSense that can be performed using a basic benchtop flow cytometer, allowing researchers to monitor key glycosylation features in near real-time, without the need for glycan release. This assay is complementary to, or may be performed prior to, traditional methods before detailed characterization is required. There are other analytical methods for monitoring of glycosylation of glycoproteins that do not require release of the glycan. For example, “top-down” mass spectrometry-based methods analyze intact glycoproteins^19,20^, as do flat or planar lectin microarrays^21,22^, which have been commercialized for detecting carbohydrates with lectins^23–26^. However, characterizing binding using suspension arrays with flow cytometry-based detection has advantages in terms of reproducibility and statistical significance when compared to the use of flat arrays and plate readers^27^. The approach does not identify individual glycans, but rather is designed to report the aggregate glycosylation features of the glycans present in a glycoprotein. This approach targets the detection of glycosylation features that are known to be critical quality attributes (CQA) in therapeutic glycoproteins^28^, such as the presence or absence of sialic acid, as well as other glycan modifications. The GlycoSense method is particularly well-suited for monitoring changes in the glycofeature composition that may occur in cell culture or during in vitro glycoengineering. Instead of generating a list of all of the glycan structures associated with a given glycoprotein, the GlycoSense method generates a “GlycoPrint” that summarizes the glycofeatures present in the intact glycoprotein analyte (Fig. 1).

**Figure 2:**
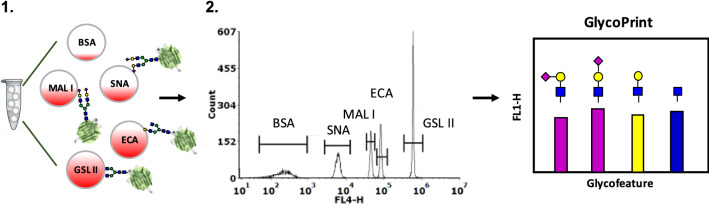
GlycoSense workflow. **1**. A fluorescently labeled glycoprotein, or glycoprotein with a fluorescently labeled secondary detection reagent, is incubated with multiplex GlycoSense microspheres in suspension then run on a flow cytometer. **2**. Microsphere peaks are determined by red (633/640 nm excitation, 670 nm emission, FL4) fluorescence while bound glycoprotein is detected by green (488 nm excitation, 525 nm emission, FL1) fluorescence. Relative median green fluorescence is then calculated for each microsphere and presented as a GlycoPrint.

In the GlycoSense approach, binding is detected between glycans and glycan-specific reagents that are conjugated to spectrally-unique microspheres (beads). By combining immobilized carbohydrate-specific reagents, such as lectins and antibodies into a multiplexed suspension array, multiple glycofeatures can be monitored simultaneously (Fig. 2). Proof of principle experiments employing bead-based multiplexing of lectins have been reported, with an emphasis on applicability to clinical diagnostics^29^. Here we broaden and generalize those initial studies to applications in glycoengineering. Moreover, by developing GlycoSense for application with commonplace benchtop flow cytometers that employ standard fluorescence detection methods, we believe that GlycoSense provides a uniquely convenient and simple tool for summarizing glycosylation features that is equally suited to a research lab environment or an industrial setting.

In a typical GlycoSense analysis, the glycoprotein analyte is incubated with a set of multiplex microspheres (discretized by their red fluorescence intensity) that contain affinity reagents specific for the glycosylation features of interest. Binding of the glycoprotein analyte to the derivatized beads is detected either by fluorescently labeling the analyte (green fluorescence) for convenience or more generally by addition of a labeled secondary detection element, such as an antibody Fab fragment. The multiplex sample is then analyzed by flow cytometry, where the microsphere type (which is mapped to the specific carbohydrate affinity reagent) is determined by its red fluorescence intensity, and analyte binding is detected by green fluorescence (Fig. 2). The green fluorescence intensity is then used to convert the signal to a GlycoPrint. Here we demonstrate that an analysis can be performed over a time course to monitor changes in the glycosylation state that occur upon sequential treatment with glycosidases. The analysis can be completed in a few minutes for each time point after an incubation period of 30–60 min, enough time for binding to reach equilibrium. In the case where an unlabeled glycoprotein is analyzed, such as might be performed during glycoprotein over-expression, the supernatant first incubated with the detection reagents, then with a fluorescently labeled secondary reagent, such as an antibody Fab fragment.

By virtue of the biosynthetic pathway, all *N*-linked glycans contain the same branched tri-mannose core, but differ in the degree to which this core has been subsequently processed by exposure to glycosidase/transferase enzymes. Thus all that is required to monitor variations in the glycosylation state is to select one or more reagent that can detect the glycofeature of interest. Typical glycofeatures that may be monitored include terminal sialic acid (Sia), D-galactopyranose (Gal), *N*-acetyl-D-glucopyranosamine (GlcNAc), D-mannopyranose (Man) and L-fucopyranose (Fuc). Moreover, with judicious selection of the affinity reagents, it is often possible to detect the linkage type and anomeric configuration associated with the glycofeature, such as Siaα2,3-, Siaα2,6-, Galβ1,4-, Galα1,3-, etc. Such linkage information is challenging to obtain in traditional glycoprofiling, requiring additional sample preparation or more specialized analytical methods^30,31^, and is frequently unreported, despite the fact that biological activity depends not only on glycan composition, but also on the linkages between residues. For example, the presence of terminal Gal in an α1,3-linkage (so called α-Gal) is known to be able to induce anaphylactic shock in humans, which has been fatal in some cases^32^.

**Figure 3:**
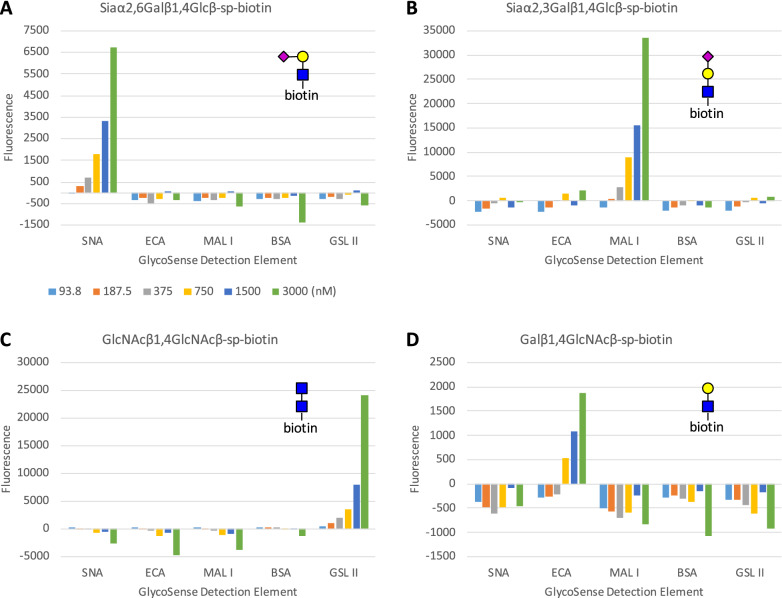
Binding of multiplex GlycoSense bead sets to biotinylated glycan standards conjugated to anti-biotin Fab labeled with DyLight 488. (**A**) SNA beads bind specifically to Siaα2,6Galβ1,4Glc. (**B**) MAL I beads bind specifically to Siaα2,3Galβ1,4GlcNAcβsp-Biotin. (**C**) GSL II beads bind specifically to GlcNAcβ1,4GlcNAc. (**D**) ECA-conjugated beads bind specifically to Galβ1,4GlcNAc. One analysis per concentration was performed for these assays. Values for sp-biotin Fab alone were subtracted from median FL1 values for each glycan Fab concentration, giving small negative values for some non-binding microspheres.

Plant lectins (also known as agglutinins) and antibodies have a long history of use as reagents in carbohydrate detection, including for the monitoring of glycofeatures^21,33–35^ and can often provide linkage information. Although lectins can have broad and sometimes complex specificities, each lectin chosen for use in this study was selected based on its well-defined specificity. In each case, the specificities were confirmed by reported glycan array data from the Consortium for Functional Glycomics (www.functionalglycomics.org), by previously reported specificity studies^36^, and by in-house quantification (Fig. 3). The detection reagents employed in this study and their specificities are presented in Table 1.

**Table 1:** Detection reagents and specificities.

Detection reagent	Terminal glycan specificity	Cognate ligand for specificity assays
*Erythrina cristagalli* agglutinin (ECA)	Galβ1,4	Galβ1,4GlcNAcβ-sp^a^-Biotin
*Maackia amurensis* lectin I (MAL I)	Siaα2,3Gal	Siaα2,3Galβ1,4GlcNAcβ-sp-Biotin
*Sambucus nigra* agglutinin (SNA)	Siaα2,6Gal	Siaα2,6Galβ1,4Glcβ-sp-Biotin
*Griffonia simplicifolia* lectin II (GSL II)	GlcNAcα/β-	GlcNAcβ1,4GlcNAcβ-sp-Biotin
Bovine serum albumin (BSA)	–	–

^a^sp = –O(CH_2_)_3_NHCO(CH_2_)_5_NH–.

## Results

### Specificity of detection elements

The specificity of each of the detection reagents presented in Table 1 was confirmed by determining their binding to fluorescently-labeled anti-biotin Fab fragments complexed to synthetic biotinylated glycans (Fig. 3). Each reagent demonstrated dose-dependent binding only to its cognate ligand, with no detectable cross-reactivity with any of the other glycans in the test set, up to a maximum glycan concentration of 3 μM. It is important to note here that even in the case that a lectin has mixed specificity, it may nevertheless be useful as a detection element, for example, GSL II is unable to discriminate between GlcNAcβ- and GlcNAcα-linkages, yet it may be effectively used to monitor the change in GlcNAc signals that occur upon treatment of a glycoprotein with galactosidase. In such an application, any cross-reactivity would be expected to remain at a constant background level during the assay.

**Figure 4:**
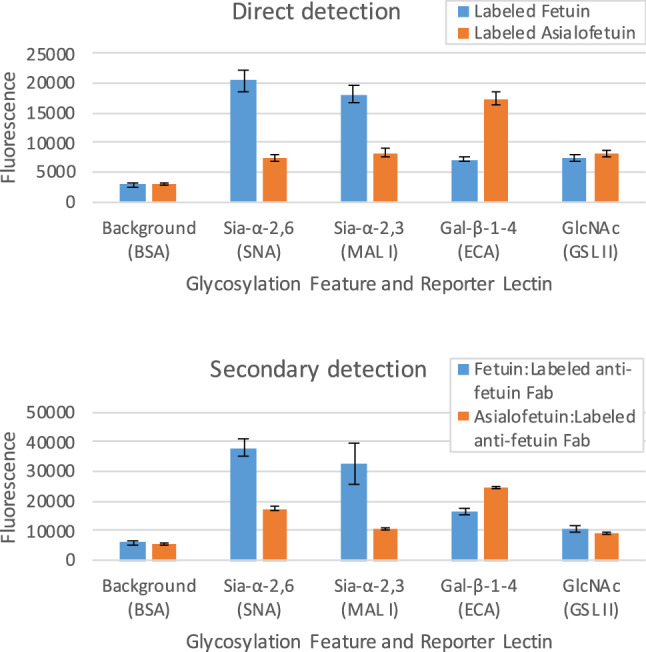
Upper: Multiplexed GlycoSense monitoring of glycofeatures using directly-labeled (SureLight 488) fetuin and asialofetuin. Lower: Indirect detection of glycofeatures employing unlabeled fetuin and asialofetuin, followed by incubation with labeled anti-bovine fetuin Fab fragment. Standard deviations from triplicates are shown as error bars.

Differences in the absolute signal intensities for each glycan detection reagent at a given analyte concentration depend on multiple factors, from variations in the level of fluorescent labelling of the anti-biotin Fab, to the binding kinetics (especially the off-rate) associated with a given lectin-glycan interaction, to the accessibility of the lectin binding sites after conjugation to the microsphere. For example, at the maximum analyte concentration (3,000 nM), MAL I gave a response (33,534 RFU) that was fivefold higher than that for SNA (6,740 RFU) each binding to its cognate glycan analyte, and 18 times higher than the signal arising from ECA (1871 RFU). It is because of these intrinsic signal differences among detection reagents that a higher signal in a GlycoPrint does not necessarily mean there is more of that terminal glycan analyte present relative to others. Nevertheless, the data from the multiplex assays (Fig. 4) show that each reagent displayed a dose dependent response only to its cognate analyte, with the lowest level of reliable signal detection in all cases being below a glycan concentration of 0.75 μM. Given the fact that the signals are dose dependent and that no cross-reactivity between detection elements was observed, the GlycoSense should be well-suited to monitoring changes in glycan composition between samples, as may arise for example during in vitro glycoengineering, in which a glycoprotein is treated with glycan-processing enzymes to modify the glycan composition.

### Direct and indirect glycoprotein labeling

Detection with the GlycoSense approach depends on the presence of a fluorescent reporter that is either directly introduced into the analyte, or formed indirectly through complexation with a labeled secondary reagent. Direct labeling is convenient when assaying purified glycoprotein samples, or for some aspects of method optimization, or when a secondary reagent is unavailable, whereas secondary detection is desirable for monitoring unlabeled glycoproteins either during glycoprotein over-expression or during in vitro glycoengineering. As seen in Fig. 4, the GlycoPrints for fetuin and asialofetuin display similar patterns, whether using direct or indirect detection methods. To eliminate the potential for glycans on the secondary reagent to compete with analyte binding, a non-glycosylated secondary reagent, such as a labeled antibody Fab fragment is preferable.

### Monitoring the treatment of a glycoprotein with sequential glycosidases

**Figure 5:**
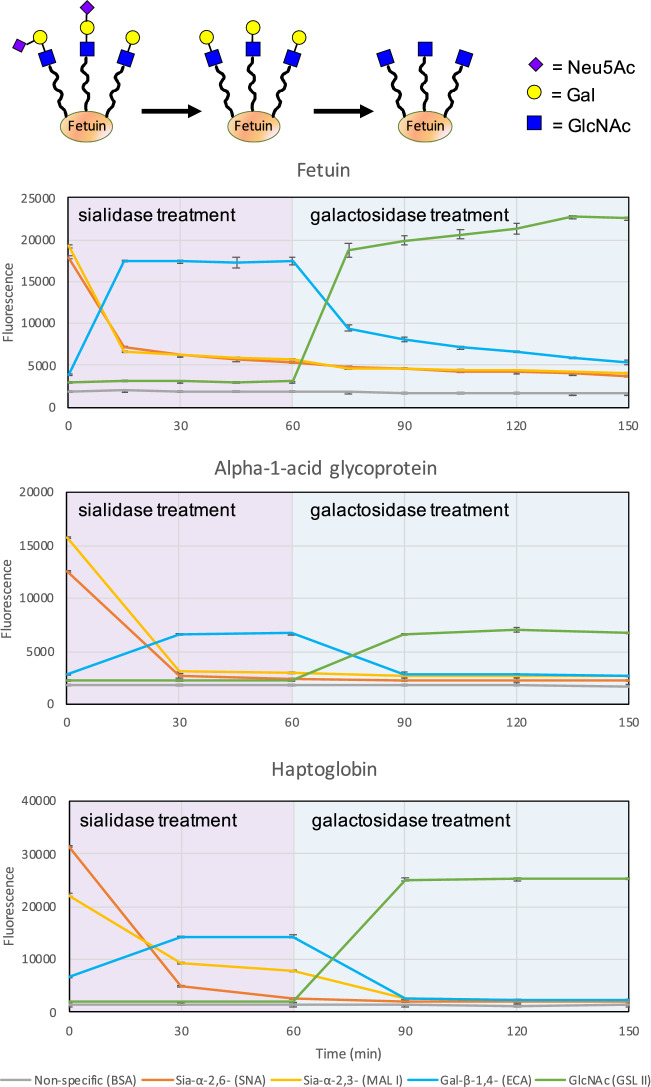
Time courses for the treatment of glycoproteins (upper panel: fetuin, middle: alpha-1-acid glycoprotein, lower: haptoglobin) with sialidase (T = 0 min) and subsequently with galactosidase (T = 60 min). Error bars are standard deviations from triplicates.

To demonstrate the utility of the GlycoSense method during in vitro glycoengineering, the fluorescently-labeled model glycoproteins fetuin, alpha-1-acid glycoprotein (AGP), and haptoglobin were treated with sialidase for 60 min, followed by treatment with galactosidase for 90 min (Fig. 5). Aliquots were collected and incubated with the GlycoSense detection elements listed in Table 1, and analyzed every 15 or 30 min over the course of the reactions, which were repeated in triplicate. An examination of the enzyme time course data in Fig. 5 indicates that the disappearance of the signal from the departing terminal monosaccharide residue is precisely paralleled by the appearance of the signal from the previously penultimate monosaccharide residue. That is, as the terminal Sia is released from its linkage to Gal upon treatment with sialidase, the signal from the Sia (orange or yellow lines in Fig. 5) decreases, while the corresponding signal from the Gal (blue lines) increases. Similarly, upon exposure to galactosidase, the Gal is released from its linkage to GlcNAc, so the signal from the Gal decreases while the corresponding signal from the GlcNAc (green lines) increases. The complementarity of the simultaneous detection of the leaving residue and the underlying residue provides a very useful corroboration of the activity and actions of each enzyme.

The rapidity of the method enables the reactions to be monitored in near real time, which provides unparalleled insight into the sensitivity of the enzyme performance as a function of the substrate composition. While sialidase treatment of AGP is fully complete within 30 min, it is still ongoing for 90 min in the case of haptoglobin, and even longer for fetuin. In the case of galactosidase treatment, AGP and haptoglobin reach completion within 30 min, while the signals from fetuin suggest that close to 90 min is required to reach an end point. Conversely, this approach provides a particularly convenient method to compare the activities of batches or samples of a given enzyme.

In order to confirm that the GlycoSense data shown in Fig. 5 arose from the expected changes in glycan structures, the experiment was repeated for fetuin with aliquots collected at the 0, 60, and 180 min time points, which were frozen to quench the enzyme reactions, and subjected to independent traditional glycoprofiling. As expected^37,38^, the glycoprofile of unmodified fetuin displayed the presence of complex sialylated glycans (Figure S1). After exposure to sialidase for one hour, the sialylated glycans were replaced by glycans carrying terminal-Gal motifs. Further treatment with galactosidases led to the formation of glycans terminating in GlcNAc, as the Gal residues were removed. Interestingly, one glycan (Figure S1 lower panel) appeared to be resistant to treatment by galactosidases. This observation is consistent with the GlycoSense analysis that indicated that the enzyme treatment was not fully complete over the course of the experiment.

### Monitoring the treatment of a glycoprotein with glycosyltransferases

**Figure 6:**
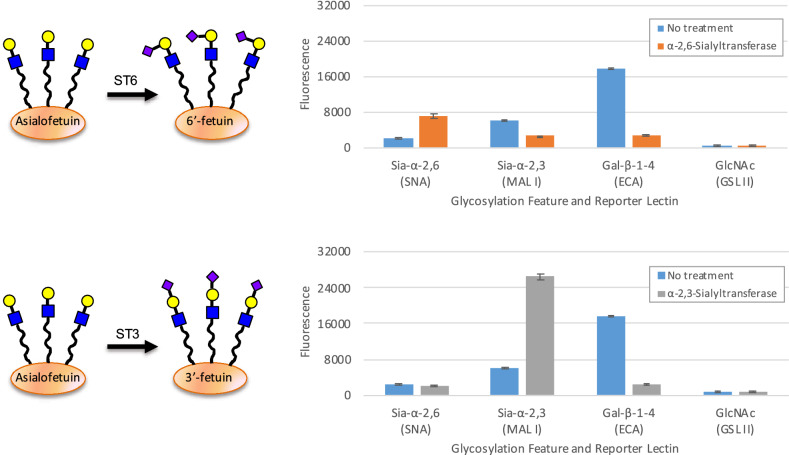
Upper: schematic representation of the glycoengineering of asialofetuin showing the effect of α-2,6-sialyltransferase (ST6) and change in glycosylation features for asialofetuin upon treatment with ST6 (orange) forming Sia-α-2,6-linkages. Lower: schematic representation of the glycoengineering of asialofetuin showing the effect of α-2,3-sialyltransferase (ST3) and change in glycosylation features for asialofetuin upon treatment with ST3 (gray) forming Sia-α-2,3-linkages. BSA background is subtracted. Standard deviations from triplicates are shown as error bars.

One key advantage of GlycoSense over traditional glycoprofiling is the ready determination of glycosidic linkage information. The nature of the linkage between sialic acid and galactose (Siaα2,3Gal versus Siaα2,6Gal) has important biological significance, for example α2,3-sialylation can destabilize antibody structure, relative to α2,6^39^. This property is significant given that Chinese hamster ovary (CHO) cells, which are frequently employed in biologics production, only form the α2,3 linkage, whereas human embryonic kidney (HEK293) cells which are also commonly employed in glycoprotein production additionally generate α2,6 linkages^40^. Despite the clear impact of these linkage differences on bioactivity, their determination remains challenging for routine mass spectrometric methods because the two sequences have identical masses. The lectins MAL I and SNA are often used to distinguish between these two linkages, for example in histochemical staining and flow cytometry experiments^41–43^, and these lectins have been incorporated in the GlycoSense suite of detection elements for this reason. To illustrate the ease with which GlycoSense can detect these linkages, fetuin lacking sialic acid (asialofetuin) was treated either with α-2,3-sialyltransferase or α-2,6-sialyltransferase, in the presence of CMP-Sia as a donor substrate, with aliquots extracted for GlycoSense analysis (Fig. 6). As in the time course analysis presented for glycosidase treatment (Fig. 5), the asialofetuin was fluorescently labeled prior to being treated with the sialyltransferases.

As expected, the transferases led to an increase binding of the GlycoSense reagents (SNA and MAL I) that detect sialic acid, and to a decrease in the signal from the Gal residues, which become inaccessible to the ECA reagent upon sialylation (Fig. 6). The magnitude of the changes in the intensities of the SNA and MAL I signals are unique to each reagent, and are therefore not directly comparable, however the decrease in the Gal signal is equivalent for both the transferase treatments because the same detection element (ECA) was employed in each case, showing that same level of sialylation has been achieved by each transferase. The GSL II probe was included as a control to indicate that no unexpected changes in the glycosylation state were occurring.

The consistently low standard deviation from replicate measurements (Fig. 6) confirms the robustness of the GlycoSense method, compared to the relatively large variations observed when plate arrays of lectins are employed in screening^44,45^. The enhanced precision of the GlycoSense method arises in part from the inherent benefits of flow cytometry (hundreds to thousands of binding events counted per aggregate GlycoPrint intensity, no washing steps, solution binding conditions), and reflects the level of consistency in the preparation of the GlycoSense detection beads.

## Discussion

Using a simple approach based on flow cytometry, changes in glycosylation features were able to be rapidly and reliably monitored during the in vitro glycoengineering of the well-characterized glycoprotein bovine fetuin. While plate-based lectin arrays have been proposed for monitoring glycosylation^46^, integration of carbohydrate detection elements into a suspension array overcomes several limitations that have hampered the wide-spread adoption of lectin arrays^47,48^. A suspension array can be multiplex, and the detection elements can be tailored to a given application on-the-fly without needing to regenerate an entire array if one element if found to have degraded, or in order to expand the array to include additional elements. Most notably, a flow-based approach results in statistical confidence in the measurement of the binding events, due to the large number of such events that are counted. Further, because the binding interaction occurs in solution, there is no need for extensive washing to remove non-specific binding.

By incorporating detection reagents specific for terminal and penultimate glycofeatures, an important source of redundancy is introduced into the GlycoSense analysis that can contribute to confidence in the resulting GlycoPrint. As a case in point, the ability to simultaneously detect both terminal sialic acid and terminal galactose residues provide orthogonal confirmation for changes arising from the loss (Fig. 5) or addition of sialic acid (Fig. 6). This is a general strategy that may be usefully exploited to detect whether a given detection reagent has degraded, or is non-functional for some reason. In the case of a flat printed array of lectins, when one detection element is no longer functional, the entire slide must be reprinted, whereas for a suspension array under those circumstances, only one set of conjugated microspheres need be replaced.

The lectins chosen in this study are known to have relatively high specificity to the terminal glycan of interest and low cross-reactivity to other terminal carbohydrates. The range of detection elements can be readily expanded to include antibodies specific to certain glycosylation epitopes (such as the Lewis blood group antigens), as well as additional specific lectins and proprietary glycan-binding molecules (Lectenz). The current limit in terms of multiplex capability is approximately 15–20 beads^49^ for a typical two-color flow cytometer. Although this diversity is significantly lower than the limits of printed (flat) arrays, it is nevertheless sufficient to permit the detection of a very wide range of glycofeatures, and, of course, the choice of detection-reagents and bead sets can be tailored to suit the specific requirements of the application.

In its current form, the GlycoSense method does not quantify the total levels of terminal glycosylation, due to challenges in accounting for the differing affinities/avidities of the detection reagents used, as well as to the dependence of the binding affinity of any given glycofeature on the individual glycan containing that feature^50^. Neither can it provide a tabulation of all of the glycans in a glycoprotein. Rather, it was developed to provide a rapid, cost-effective, easy to use alternative to complete glycoprofiling that provides a reliable overview of aggregate glycofeatures. Two-laser flow cytometers are common in academic and industrial laboratories, significantly reducing the cost of such an analysis, and additionally, flow cytometers do not require dedicated personal or in-depth training to operate, making this platform convenient for use both at-line during glycoprotein production, as well as in a research environment. Additionally, many organizations have core facilities with open-access to multiple flow cytometers. A GlycoSense analysis is not intended to, nor is it able to, provide the same information as traditional glycoprofiling methods. Nonetheless, it is well suited to monitoring changes in key glycosylation elements over a time course, such as during glycoengineering, where it provides a rapid GlycoPrint of key glycosylation patterns. Such a near real time approach offers many potential benefits for the production of biologics and biosimilars, including the screening of production conditions with regard to generating a glycoprotein with glycofeatures that match a desired profile. Additionally, a rapid assessment of the glycosylation state could provide time for corrective measures to be taken in the event that aberrant glycosylation patterns were detected during production. Lastly, the GlycoSense method could be used to measure glycoprotein quality, for example after prolonged storage, or to detect differences in glycosylation between batches. Continued work on this approach will include its application in glycoprotein production, as well as in the development of secondary detection methods, and the creation and characterization of additional glycoprotein standards.

## Methods

### Protein sources

All lectins listed were purchased from Vector Laboratories (Burlingame, CA). BSA was purchased from Thermo Fisher Scientific (Waltham, MA). Bovine fetuin, asialofetuin, and native human alpha-1-acid glycoprotein were purchased from Sigma-Aldrich (St. Louis, MO). Native human haptoglobin was purchased from Bio-Rad (Hercules, CA). Anti-bovine fetuin polyclonal antibody was purchased from GeneTex, Inc. (Irvine, CA). Papain coated magnetic particles were from Spherotech (Lake Forest, IL). Sialidase A 66 was purchased from Agilent (Santa Clara, CA) while β1-3 Galactosidase and β1-4 Galactosidase S were purchased from New England Biolabs (Ipswitch, NH). Additional β1-4 Galactosidase used on human haptoglobin and alpha-1-acid glycoprotein was purchased from Agilent. Alpha-2,6-Sialyltransferase, rec. and α-2,3-Sialyltransferase, rec. were purchased from Roche Diagnostics GmbH (Mannheim, Germany).

### Microsphere conjugation

Approximately 5 × 10^6^ of each 5–5.9 µm microsphere from SPHERO Carboxyl Particle Kit (Spherotech, Lake Forest, IL) were aliquoted per conjugation reaction into copolymer tubes (USA Scientific, Ocala, FL) and washed with water, then 100 mM sodium phosphate pH 6.2 washing by centrifugation for 2 min at maximum speed in a Fisher Scientific accuSpin Micro 17 centrifuge and removal of supernatant after microsphere pelleting. 5 mg/mL *N*-hydroxysulfosuccinimide (Sulfo-NHS, Thermo Scientific, Waltham, MA) and 5 mg/mL 1-ethyl-3-(3-dimethylaminopropyl)carbodiimide hydrochloride (EDC, Thermo Scientific, Waltham, MA) were added to microspheres for activation and microspheres were incubated for 20 min in suspension. Two washes of 50 mM MES pH 5.0 (Sigma-Aldrich, St. Louis, MO) were performed.

Conjugation was performed in 50 mM MES pH 5.0 with 0.2 mg/mL protein conjugate incubated with activated microspheres for 2 h. After centrifugation and removal of protein solution, unreacted activated carboxyl groups were quenched using 0.15 M hydroxylamine hydrochloride (Sigma-Aldrich, St. Louis, MO) incubating for 30 min in suspension. Two separation and wash steps were performed using Phosphate Buffered Saline (PBS, Amresco, Solon, OH) with 0.025% Tween-20 (Amresco, Solon, OH) and 0.05% sodium azide (Sigma-Aldrich, St. Louis, MO). Conjugated microspheres were pelleted by centrifugation and suspended in 250 µL of PBS with 0.025% Tween-20 and 0.05% sodium azide and stored at 4 °C.

### Glycoprotein labeling/secondary detection

Glycoproteins or secondary detection reagents were labeled with DyLight 488 NHS ester (Thermo Scientific, Waltham, MA) or SureLight 488 NHS ester (Columbia Biosciences, Frederick, MD) according to the manufacturers’ instructions, and free dye was removed using Pierce Dye Removal Columns (Thermo Scientific, Waltham, MA). Labeled glycoprotein was quantified using a NanoDrop 2000, measuring absorbance at 280 nm and 493 nm and a molar extinction coefficient calculated based on sequence information.

### Biotinylated glycan binding assays

All biotinylated glycans used in glycan binding experiments were purchased from Glycotech (Gaithersburg, MA). Synthetic biotinylated glycan were incubated with goat anti-biotin Fab polyclonal antibody (Rockland Immunochemicals, Pottstown, PA) labeled with DyLight 488.

Biotinylated glycan was bound to dye labeled anti-biotin Fab at a concentration of 6 uM at a 1:1 ratio for 1 h covered from light in PBS + 1 mg/mL bovine serum albumin (BSA, Sigma, St. Louis, MO). Conjugated microspheres were pipeted into PBS with 1 mg/mL BSA with approximately 15,000 of each of the multiplex microspheres conjugated with lectins per 50 μL volume. Serial dilutions of 6 μM glycan/Fab were performed by diluting 50 μL into 50 μL of PBS + 1 mg/mL BSA, with 50 μL of GlycoSense microsphere mix added to each 50 μL dilution of glycan/Fab for final concentrations of 3,000 nM, 1,500 nM, 750 nM, 375 nM, 189.5 nM and 93.75 nM glycan/Fab. Glycan/DyLight 488 labeled Fab was incubated with GlycoSense beads for 1 h on a rotator device covered from light, then run on an Accuri C6 flow cytometer (BD Biosciences, Franklin Lakes, NJ) on slow speed, collecting at least 1,000 of each microsphere per sample. Microspheres were gated by size on a bivariate dot pot of forward scatter (FSC) and side scatter (SSC) with a threshold cutoff of 80,000 on FSC. An FL4-H channel (633 nm excitation, 675/25 nm emission) histogram plot on FSC/SSC size gated events was created with individual microsphere gates drawn on FL4-H peaks. FL1-A (488 nm excitation, 533/30 nm emission) histogram plots for each microsphere, gated on the FL4-H histogram plot, were created with median FL1-A channel (488 nm excitation, 533/30 nm emission) fluorescence values for each microsphere calculated. No compensation was used in any of the experiments as there is no overlap between the bead (FL4) fluorescence and the DyLight 488 (FL1) labeled protein fluorescence.

A control experiment, with the sp-biotin linker purchased from GlycoTech, bound at a 1:1 ratio over the same concentration range, was performed for each batch of anti-biotin Fab used, with median FL1-A values of the sp-biotin linker subtracted from the glycan/Fab median FL1-A values for each concentration. Data analysis was performed using FCS Express 4 (De Novo Software, Los Angeles, CA) and a custom-made template for measuring median FL1-A fluorescence from each FL4-H microsphere peak.

### Glycoprotein binding and cytometry measurements

Glycoprotein binding to microspheres was performed using 1 μM labeled (or unlabeled) protein in 100 μL volumes with approximately 10,000 to 20,000 of each conjugated microsphere present in 10 or 50 mM HEPES, 10 mM NaCl, 1 mg/mL BSA pH 7.4. Glycan titrations in Fig. 3 and fetuin, asialofetuin, human haptoglobin, and alpha-1-acid glycoprotein experiments were bound to microspheres in phosphate buffered saline (PBS) with 1 mg/mL BSA. Incubations were performed for 1 h at room temperature on a rotator suspension device with samples protected from light.

For detection with a labeled secondary reagent (anti-bovine fetuin polyclonal antibody treated with papain magnetic particles to generate the Fab fragment), the samples were centrifuged, the supernatant removed, and 1.5 μM labeled anti-bovine fetuin Fab in 100 μL was added to the analyte-microspheres. Incubations were performed for an additional 1 h at room temperature on a rotator suspension device with samples protected from light.

Samples were run on a BD Accuri C6 flow cytometer collecting at least 2,000 of each microsphere present. Median FL1-A values were calculated from each FL4-H histogram peak using the same FCS Express 4 template described above.

### Sialidase and galactosidase treatment

A 5 μM stock of glycoprotein was prepared in an optimized glycan modification buffer based on Prozyme/Agilent β1-3,4 galactosidase buffer (30–40 mM sodium citrate, 60–70 mM sodium phosphate (made based on personal communication with Prozyme/Agilent) with 1 mM calcium chloride added and pH raised from 4.0 to 5.5. Beta 1–3 Galactosidase and β1-4 Galactosidase S) galactosidase activity was tested in this buffer using 4-methylumbelliferyl-α-D-galactopyranoside (Sigma-Aldrich, St. Louis, MO), while Sialidase A 66 activity in this buffer was tested using 2′-(4-Methylumbelliferyl)-α-D-N-acetylneuraminic acid sodium salt hydrate (Sigma-Aldrich, St. Louis, MO). Fetuin was incubated at 37 °C for 15 min, and triplicate GlycoSense microsphere measurements were taken. 0.025 Units of Sialidase A 66 (a unit is defined as the amount which cleaves 1 µmole of p-nitrophenol from p-nitrophenyl-α-D-N-acetylneuraminic acid per minute at 37 °C, pH 5.5) was added to the fetuin mixture, which was incubated at 37 °C in a water bath, and triplicate GlycoSense measurements were taken every 15 min. After 1 h of Sialidase A 66 treatment 10 Units of β1-3 Galactosidase (New England Biolabs, Ipswitch, MA) and 10 Units of β1-4 Galactosidase S (New England Biolabs, Ipswich, MA) were added to the fetuin, which was incubated at 37 °C in a water bath, with triplicate GlycoSense measurements taken every 15 min. (One unit is defined as the amount of enzyme required to cleave > 95% of the terminal, β-D-galactose from 1 nmol Galβ1-4GlcNAcβ1-3Galβ1-4Glc-7-amino-4-methyl-coumarin (AMC), in 1 h at 37 °C in a total reaction volume of 10 μL.) NEB and Agilent enzyme units are defined differently and optimal amounts and incubation times were determined experimentally. A 2.5 μM stock of Surelight 488 labeled haptoglobin was prepared in 1X Agilent Reaction buffer B with triplicate 250 nM samples taken and bound to microspheres prior (time 0) prior to addition of 0.025 Units of Agilent Sialidase A 66, with triplicate 250 nM measurements bound to microspheres every 30 min. After 1 h, 0.01 Units of Agilent β1-4 galactosidase was added, and triplicate 250 nM measurements were taken every 30 min. Surelight 488 labeled alpha-1-acid glycoprotein was treated in the same way as haptoglobin with a starting stock of 5 μM protein, and 500 nM sample was bound to microspheres.

An additional sialidase and galactosidase time course experiment was repeated using 5 μM SureLight 488 labeled fetuin in the optimized glycan modification buffer described above, taking triplicate GlycoSense measurements at 0 min, 60 min, and 180 min time points, with 200 μL of fetuin removed and frozen at each time point. 0.025 Units of Sialidase A 66 were added after the 0 min time point, and 10 Units of β1-3 Galactosidase and 10 Units of β1-4 Galactosidase S were added after the 60 min time point. Fetuin was incubated at 37 °C during the course of the experiment. Frozen samples were submitted to the University of Georgia Complex Carbohydrate Research Center and release and permethylation of *N*-linked glycans was performed on each time point to confirm glycan modifications monitored by the GlycoSense method were present (Supplementary Information). Whole amount of all three sample was taken for *N*-glycan profiling. The sample was reduced and alkylated using DTT and iodoacetamide and then *N*-glycans were released by enzymatic cleavage with PNGase F at 37 °C overnight. *N*-glycans were purified of any contaminants with a C18 sep-pak cartridge. The carbohydrate fraction was eluted with 5% acetic acid and dried by lyophilization. Released *N*-linked oligosaccharides were permethylated by using methyl iodide on DMSO/NaOH mixture. The glycans were dried with nitrogen gas and profiled by MALDI-TOF analysis.

### Sialyltransferase treatment

Two samples of 5 μM SureLight 488 labeled asialofetuin were set up in a sialyltransferase buffer of 100 mM MES (Sigma-Aldrich, St. Louis, MO) and 500 μg CMP-NANA (Roche, Basel Switzerland) pH 6.5. 200 μM ZnCl_2_ (Sigma-Aldrich, St. Louis, MO) and 2 μg alkaline phosphatase (Roche, Basel, Switzerland) were added to prevent reverse reactions according to the provided protocol for sialyltransferase. 56 μg of α-2,6-sialyltransferase was added to one sample while 56 ug of α-2,3-sialyltransferase was added to the other. Samples were incubated at 37 °C for two hours in a water bath. Triplicate samples of 1 μM both sialyltransferase treated asialofetuin samples as well as triplicate 1 μM untreated asialofetuin were measured by GlycoSense with median FL1 values for each bead calculated in the manner described above.

## Supplementary Information

Supplementary Information.

## Data Availability

The datasets generated during and/or analyzed during the current study are available from the corresponding author on reasonable request.
