# Supplementary material for: Simplifying the detection and monitoring of protein glycosylation during in vitro glycoengineering
Source: Sci Rep. 2023 Jan 11;13:567. doi: 10.1038/s41598-023-27634-z (PMC9834283; doi:10.1038/s41598-023-27634-z)
Supplement: Supplementary file 1 — Supplementary Information. [file 41598_2023_27634_MOESM1_ESM.pdf]

Simplifying the detection and monitoring of protein glycosylation during in vitro glycoengineering

Matthew J. Saunders[1], Robert J. Woods[2] and *Loretta Yang[1]
[1]Lectenz Bio, 111 Riverbend Rd, Athens, GA 30602
[2]Complex Carbohydrate Research Center, University of Georgia, 315 Riverbend Rd, Athens, GA 30602
*Corresponding author

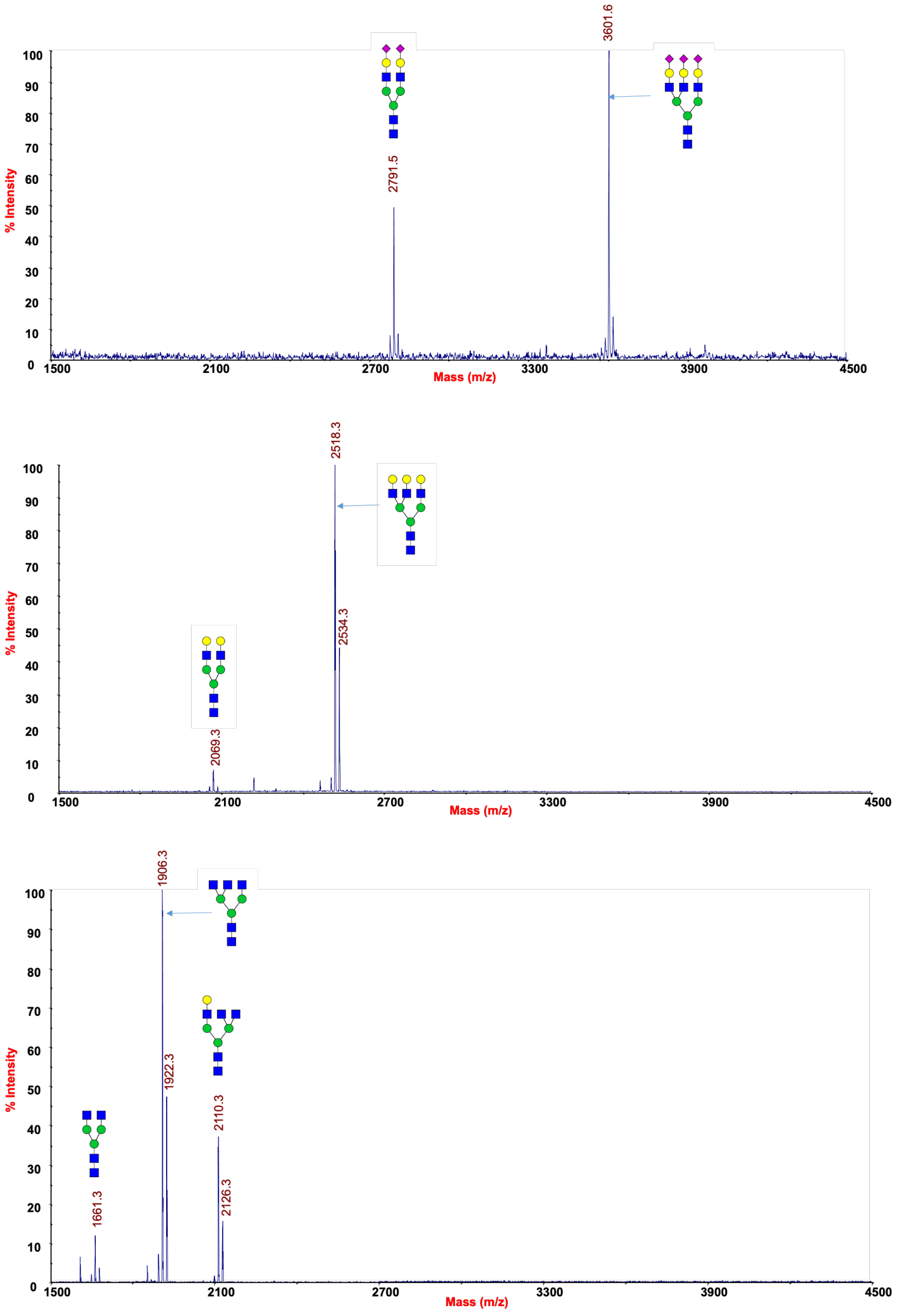

Figure S1. *N*-glycans released from fetuin sequentially treated with first Sialidase A and subsequently with β1,3-Galactosidase and β1,4-Galactosidase S, determined using MALDI-TOF, corresponding to the t = 0 (upper), 60 (middle), and 180 minute (lower) time points in Figure 5 (upper panel).
